# Dietary and Nutritional Strategies for Patients with Glioma: A Narrative Review of Treatment, Recovery, Immune Support, and Microbiota Modulation

**DOI:** 10.3390/nu18060975

**Published:** 2026-03-19

**Authors:** George B. H. Green, Alexis N. Cox-Holmes, Jonathan T. Flowers, Michael B. Williams, Anna Claire E. Potier, Jeri L. Brandom, Stephen A. Watts, Raymond Luke, Jennifer S. Yu, Braden C. McFarland

**Affiliations:** 1 Department of Cell, Developmental and Integrative Biology, University of Alabama at Birmingham, Birmingham, AL 35294, USA; 2 Department of Biology, University of Alabama at Birmingham, Birmingham, AL 35294, USA; 3 Department of Radiation Oncology, Weill Cornell Medical College, New York, NY 10065, USA; 4 Department of Radiation Oncology, Department of Cancer Biology, Cleveland Clinic, Cleveland, OH 44195, USA

**Keywords:** glioma, nutrition, microbiota, surgery, wound healing

## Abstract

This narrative review aims to explore the relationship between glioma and nutrition throughout stages of treatment and recovery. Gliomas are aggressive brain tumors that significantly impair quality of life and present treatment challenges. There has been a growing interest regarding the gut–brain axis and the microbiome, particularly their roles in modulating immune function and influencing the response to cancer treatment. This review examines how specific nutritional approaches may assist patients throughout the course of chemotherapy, radiation, immunotherapy, surgical intervention, and the recovery process. It also addresses the potential for integrative nutritional approaches to complement conventional treatment and improve clinical outcomes. Emerging evidence suggests that nutrition may influence immune function, treatment-related side effects, and the tumor microenvironment, in part through effects on the gut microbiota. Nutritional support during therapy has been linked to increased strength, decreased inflammation, and improved treatment tolerance. Dietary patterns may influence gut–brain interactions and systemic immune responses, opening the potential to improve therapeutic outcomes in glioma. In summary, nutrition may represent an important supportive component of glioma care, while microbiota-mediated and metabolic dietary strategies remain areas of active investigation. Further clinical studies are needed to determine whether specific nutritional interventions can improve survival, treatment response, or quality of life in patients with glioma.

## 1. Introduction

Gliomas, tumors originating from glial cells in the brain, are the most common type of primary tumor found within the central nervous system (CNS) [1]. These tumors account for 25% of all brain tumors and 80% of all malignant brain tumors, with the most aggressive being glioblastoma (GBM). Gliomas are categorized based on the phenotypic characteristics of the cells of origin—oligodendroglioma, ependymoma, or astrocytoma—as well as by grade, grades 1–4, and molecular features: for example, isocitrate dehydrogenase (IDH) mutation status or histone H3K27 mutation status [2,3]. As the glioma becomes more aggressive, the prognosis for the patient rapidly declines. Currently, the median survival for patients with GBM at the time of diagnosis is 15–16 months [4].

Therapeutic options depend on the location and grade of the tumor; however, treatment often consists of surgical resection and subsequent chemotherapy and radiation. Surgery is typically the primary treatment for brain tumors as it establishes a diagnosis and relieves mass effect. The use of adjuvant treatments such as chemotherapy and radiation is considered based on the risk of recurrence. For example, for patients with GBM, concurrent radiation and chemotherapy followed by maintenance chemotherapy are the standard of care. Tumor treating fields (TTFs, Optune) may also be employed [5]. Immunotherapy has shown promising results in other cancers but has not been successful in treating gliomas potentially due to the immunologically privileged environment of the CNS, heterogeneity, low mutational burden of the tumor, and associations with the gut microbiome [6]. Despite the intensive therapy regimen, treatment options are often insufficient and prognosis for patients diagnosed with these malignant brain tumors is typically poor due to the invasive nature of the tumors.

A promising area of study for the treatment of glioma patients involves dietary or nutritional therapies. Diet may influence an individual’s risk for developing cancer, but novel research supports the positive impact of proper nutrition on survivorship [7]. Modifying nutritional intake could improve the quality of life and treatment outcomes for cancers with unfavorable prognoses, such as GBM [8].

This narrative review summarizes the current evidence on nutrition, metabolism, and the gut microbiota in glioma, with an emphasis on how nutritional considerations may differ across phases of care including perioperative management, chemotherapy, radiation, immunotherapy, and recovery. We also distinguish between preclinical, observational, and clinical evidence to clarify the translational relevance of the field.

## 2. Clinical Context: Glioma Treatment Course and Associated Nutritional Challenges

### 2.1. Surgical Resection and Perioperative Nutritional Vulnerability

Surgery is one of the primary treatment options for patients with glioma, and the goal is to prevent tumor progression and further damage to the brain while maintaining neurological function [9]. Although surgical resection can improve a patient’s prognosis, glioma recurrence is almost always inevitable due to the aggressive nature of the cancer [10]. Research has shown that, since over 50% of GBMs are in vital areas of the CNS, a total gross total resection can also lead to severe neurological injury and deficits [9]. For patients in whom <80% of tumor resection is possible, biopsy is preferred, followed by definitive radiation and chemotherapy [11].

Recovery from glioma resection surgery may lead to malnutrition due to tumor-associated factors. This malnutrition can impair the body’s ability to tolerate subsequent anti-tumor therapies and increase the risk of adverse reactions [12]. In one study, glioma patients receiving individualized nutritional management following surgery had higher levels of albumin (ALB), prealbumin (PA), and hemoglobin (Hb) compared to patients receiving routine dietary care. These biomarkers are commonly used indicators of nutritional status and protein synthesis. Fourteen days postoperatively, patients receiving targeted nutritional support showed improved nutritional markers, suggesting that structured nutritional management during recovery may reduce the risk of malnutrition and improve treatment tolerance [12].

### 2.2. Chemotherapy and Metabolic/Nutritional Burden

Chemotherapy is often given for patients with aggressive gliomas. Common chemotherapies used for adult patients with gliomas include temozolomide (TMZ) or procarbazine, lomustine, and vincristine (PCV) [13]. TMZ is an alkylating agent that crosses the blood–brain barrier and is frequently administered in combination with radiotherapy. The MGMT promoter methylation status is commonly used as a biomarker to predict responsiveness to TMZ. In some cases, tumor treating fields may also be used alongside TMZ therapy [14,15,16].

Chemotherapy can contribute to metabolic and nutritional disturbances through several mechanisms. Treatment-related lymphopenia may result in immunosuppression and an increased susceptibility to infections [17]. Alkylating agents such as TMZ, used for patients with glioma, are filtered through the kidneys, and may lead to nephrotoxicity. Due to the metabolic changes and potential toxicities caused by chemotherapeutics, a patient undergoing this treatment likely will experience decreased energy intake and cancer-related malnutrition, physical inactivity, and decreased muscle function [18]. Patients with cancer experience weight fluctuations and nutritional deficiencies due to an imbalance between energy intake and total daily energy expenditure (TEE) [19].

Nutritional support during chemotherapy has been associated with improved tolerance to treatment and a reduced incidence of complications such as nausea, mucositis, and gastrointestinal distress in oncology populations [20]. However, most evidence regarding dietary strategies in glioma patients specifically remains limited, and many proposed nutritional interventions are based on the broader oncology literature or preclinical studies. Therefore, nutritional management during chemotherapy in glioma should primarily be considered supportive care aimed at maintaining energy balance, preserving lean mass, and improving treatment tolerance.

### 2.3. Radiation Therapy and Nutritional Consequences

Radiotherapy utilizes radiation targeted to very specific regions of the tumor or tumor bed and areas of presumed tumor infiltration. Typically, after healing from surgery, patients with grade 2–4 gliomas will undergo approximately 6 weeks of radiation. Radiation kills tumor cells by damaging DNA and slowing the growth of cancerous cells. Evidence from animal models has shown that radiation targeted to areas of the CNS caused damage to multiple types of neural cells, which led to vascular damage in the brain and the impairment of glial cell function. These changes caused reduced neurogenesis in the hippocampus, ultimately leading to neural deficits and an increased chance for neuroinflammation from activated microglia [21]. Alterations like this can contribute to a patient developing radiation-induced cognitive dysfunction, again decreasing their quality of life.

Ionizing radiation treatment for cancer cells can also elicit indirect damage to healthy cells. Radiation-induced reactive oxygen species (ROS) can cause damage to the macromolecules in the cell and lead to oxidative stress and the disruption of redox homeostasis [22]. These physiological stresses, combined with treatment-related fatigue, nausea, and vomiting, may reduce dietary intake and contribute to malnutrition, sarcopenia, and cachexia in some patients [23]. In severe cases, dehydration and electrolyte imbalances may occur, which can negatively affect quality of life and delay the continuation of therapy [24]. Despite these potential nutritional challenges, relatively few studies have specifically examined dietary or nutritional interventions during radiation therapy in patients with glioma [23].

### 2.4. Corticosteroids and Metabolic Complications

The use of dexamethasone to reduce cerebral edema post-operatively and during radiotherapy can also alter metabolism and cause hyperglycemia, fluid retention, and weight gain [25]. In the long term, the use of steroids can contribute to fat accumulation and redistribution and proximal muscle wasting [26]. The impairment of the hypothalamic–pituitary axis may also cause hormonal imbalances such as hypothyroidism that further exacerbate metabolic disturbances [27,28].

### 2.5. Immunotherapy and Emerging Nutritional Considerations

Immunotherapies work by engaging the patient’s immune system to recognize abnormal, cancerous cells and eliminate them from the body. There are various types of immunotherapies used for treating glioma, and they include immune checkpoint blockades (ICBs), chimeric antigen receptor (CAR) T cell therapies, oncolytic viruses, and vaccines [29]. Although these approaches have shown promise in some malignancies, their clinical benefit in glioma remains limited and is an area of ongoing investigation.

Immunotherapy is associated with a range of potential adverse effects. Immune-related toxicities may involve multiple organ systems and can include gastrointestinal symptoms such as diarrhea, as well as endocrine disturbances including pancreatic dysfunction, altered glycemic control, and in rare cases diabetic ketoacidosis [30].

The adverse effects of immunotherapy can lead to malnutrition and may negatively affect treatment tolerance and overall clinical outcomes. The likely reason for malnutrition with immunotherapy is due to reports of nausea, diarrhea, fatigue, and decreased appetite, with these symptoms being more frequent for patients with later-stage cancer and on higher doses of the treatment [30]. To our knowledge, dietary or nutritional interventions have not been adequately investigated in response to immunotherapy.

## 3. Evidence-Based Nutritional Considerations in Glioma

### 3.1. Carbohydrates, Glycemia, and Metabolic Context

Carbohydrate intake and glycemic regulation represent important metabolic considerations for patients with cancer. Many tumors, including gliomas, exhibit metabolic reprogramming characterized by increased glucose uptake and a reliance on aerobic glycolysis, commonly referred to as the Warburg effect [31]. Rather than a preference, gliomas become metabolically dependent on glucose-fueled lactic acid fermentation due to insufficient energy production via oxidative phosphorylation, making glucose metabolism a requirement for growth and survival. This dependency has led to dietary strategies aiming to restrict carbohydrate intake to reduce glucose availability, thereby potentially impairing tumor viability and limiting growth [32,33,34,35]. This is especially important for patients with hyperglycemia who already have difficulty maintaining a healthy blood glucose level. Montemmo et al. observed that, in patients with glioma, hyperglycemia was a risk factor for poorer overall survival (OS) independent of being diagnosed with DM2 [36,37].

Patients with glioma and DM2 receiving metformin to assist with blood glucose regulation had an increase in OS by several months. Lu et al. also observed that hyperglycemia in patients with glioma was an independent risk factor that resulted in poorer OS [36,37]. The average prevalence of DM2 in all study cohorts in the Lu et al. meta-analysis was 9%, which is close to the prevalence in the American population in general at 8.9% [37,38]. Many of the recommended nutrients discussed below help maintain a low postprandial blood glucose that is near resting measures. Nutritional approaches that aim to reduce glycemic load, such as limiting highly refined carbohydrates or high-glycemic-index foods, have therefore been proposed. However, the clinical efficacy of carbohydrate-restricted dietary strategies in glioma patients remains uncertain and requires further investigation.

### 3.2. Protein Intake and Preservation of Lean Mass

Of the three macronutrient classes, dietary protein has the most significant impact on lean mass maintenance and growth, and an insufficient intake leads to muscle wasting and frailty, especially in compromised patients [39,40]. Dietary protein provides both a source of energy and building blocks for tumor growth directly or indirectly from the conversion of glycolytic amino acids to glucose [41]. Boutiere et al. recently highlighted that, although reports of impacts of dietary protein amount in cancer patients are limited, experts still recommend an increased protein intake for patients with cancer over the general recommendations for the American population [42]. Newly diagnosed patients with glioma exhibited muscle loss, indicating that muscle atrophy may occur in patients independent of chemotherapy or radiological treatments [43,44].

Cho et al. supplied brain tumor patients undergoing rehabilitation either a small amount of dietary protein or a placebo of carbohydrates for six weeks, resulting in an increased pinch grip strength and increased six-minute walking distance for the protein-supplemented group [45]. Patients in the protein-supplemented group that exhibited moderate malnutrition had a significant increase in fat-free mass and skeletal mass at the end of the six-week period. Studies evaluating temporal muscle thickness, a proxy for skeletal muscle mass, show a better OS with higher thickness, suggesting that lean-mass body content promotes more favorable outcomes for patients [46,47]. Increased dietary protein may help mitigate muscle loss and frailty in patients with cancer, which may support functional status and treatment tolerance.

### 3.3. Lipids and Inflammatory Balance

Lipids serve as energy sources, cell components, and essential signaling molecules [48]. Dietary lipids play a role in cancer prevalence and metabolic disease states such as systemic inflammation, which are associated with different types of cancers. Research suggests that saturated fats and ω-6 polyunsaturated fatty acids (PUFAs) from dairy and terrestrial animal sources are more pro-inflammatory and that ω-3 PUFAs from unrefined oils (olive oil, avocado oils, etc.) and marine animal sources (marine fishes and crustaceans) are more anti-inflammatory [49]. A review of the literature surrounding dietary ω-3 PUFAs suggests that the reduced incidence of cancer is attributed to anti-inflammatory and anti-free radical mechanisms [50]. A meta-analysis showed that saturated fat intake was linked to increased incidences of breast, prostate, and colorectal cancers [51]. However, a meta-analysis evaluating ω-3 PUFAs found that breast cancer instances were reduced and that OS for cancers overall were increased [52,53].

There are a few reports on the impact of dietary lipids on patients with glioma and glioma formation. Brain tissue has a high lipid content and high dietary lipid demand during development, which is typically met by the specific placenta fatty acid transport during pregnancy and breast feeding after birth, as milk contains approximately 60% of its calories from fat and essential fatty acids for growth [54,55,56]. As brain tumors develop, they also utilize a large quantity of fatty acids from dietary sources and from de novo synthesis, indicating the specific importance for glioma-specific recommendations for lipid intake [57]. One case–control study specific to glioma reports that persons in the highest quartile for PUFA intake had a lower instance of glioma formation, and this benefit extended to those at a higher body mass index [58]. This study does not, however, differentiate between different types of PUFAs that may be pro- or anti-inflammatory. Another case–control study that examined the dietary inflammatory score showed that glioma risk increased with a more inflammatory diet; however, that score is a combination of several factors, and dietary PUFA and ω-3 PUFA intake was only a part of the measurement [59].

Given the limited glioma-specific evidence, the current dietary recommendations regarding lipid intake in glioma patients are largely extrapolated from broader nutritional research. Diets emphasizing unsaturated fats and anti-inflammatory lipid sources may represent a reasonable general approach, although further clinical studies are needed.

### 3.4. Micronutrients and Bioactive Compounds

Micronutrients are nutrients required in milligrams per kilogram of body weight for normal physiological functions, which contribute to antioxidant, antiangiogenic, and pro-autophagy responses. The intake of antioxidants from several sources, calculated as either the Total Antioxidant Capacity or Dietary Antioxidant Index, shows a decreased incidence of glioma and improved OS for patients with glioma [60,61].

Meta-analyses of antioxidant vitamins showed that vitamins C and A were associated with decreased incidences of gliomas, but vitamin E was not [62,63,64,65,66]. However, DeLorenze et al. showed that outcomes related to antioxidant consumption varied greatly between histological glioma groupings, with a high intake of some antioxidants only showing OS benefits for lower-grade gliomas, while having a negative impact on OS in higher-grade gliomas [67]. These data suggest that recommendations for antioxidant levels or ratios could be based on glioma type and development, as some studies that did not group patients into histological categories may have missed instances of negative antioxidant impacts.

A number of dietary flavonoid and phytochemicals have antiangiogenic and neuroprotective effects, making them areas of ongoing investigation in glioma research [68,69]. A meta-analysis of glioma mouse and rat models showed that the flavonoid resveratrol, a bioactive food component, was strongly associated with anti-aging effects, the metabolic rate, and slowed tumor growth, and increased the effectiveness of TMZ [70]. A prospective study found an inverse relation between the total dietary flavonoid intake and glioma incidence [71]. Vitamins are another micronutrient that may be important for patients with glioma. Takahashi et al.’s meta-analysis found no relation between serum vitamin D (25(OH)D) levels and glioma in general [72]. They did, however, find a reduction in GBM, the most aggressive glioma subtype, for those with higher serum vitamin D. A meta-analysis examining both cell lines and animal model glioma found that cell and tumor growth is suppressed by vitamin D and that autophagy was activated [73].

## 4. Dietary Patterns and Metabolic Strategies in Glioma

Several dietary patterns have been proposed to influence cancer metabolism, inflammation, or systemic metabolic health. While direct clinical evidence in glioma patients remains limited, several dietary approaches have been explored in preclinical models or broader oncology populations. These include caloric restriction strategies, low-carbohydrate or ketogenic diets, and dietary patterns emphasizing minimally processed foods and anti-inflammatory nutrient profiles [74]. There are also recommended diets suggesting alternative combinations of these nutrients such as lower-calorie, high-fat diets or Paleolithic–ketogenic diets [75].

### 4.1. Caloric Restriction and Fasting

Low-calorie diets or caloric restriction (CR) diets aim to reduce tumor access to nutrition to a level that slows growth or to an extent that induces autophagy and can either be temporary or long-term [76]. It has also been documented that a period of fasting makes tumors more sensitive to chemical and radiological therapies in patients with glioma and other cancers [77,78]. Mahlke et al. provided mice with carcinogen-induced glioma (ethylnitrosourea) with a diet *ad libitum* or with a CR diet (40% of the calories of the *ad libitum* intake) [79]. Mice that received the CR diet had decreased glioma numbers in brain sections and a decreased average glioma size at 8 weeks, and markers for oxidative stress and anti-tumor growth were all changed in a beneficial direction for CR mice. Jiang et al. provided mice transplanted with glioma cells with a diet *ad libitum* or a CR diet (40% of the calories of the *ad libitum* intake) and found reduced tumor growth and decreased edema [80]. A meta-analysis looking at animal models of cancer for CR and intermittent fasting showed that CR decreased the odds ratio for multiple types of cancer, but intermittent fasting did not impact cancer onset [81].

### 4.2. Ketogenic and Low-Carbohydrate Diets

Low-carbohydrate diets are often represented as the Atkin’s diet and ketogenic diet (KD). Carbohydrate intake in the Atkin’s diet is restricted only in the initial phases. Keto adaptation represents a shift from glucose metabolism to fat metabolism, and ketone bodies are utilized as the major energy source later in KD. KD has been around for over 100 years, since it was first used as an alternative treatment to fasting for epileptic patients and has more recently had a resurgence in popularity as a weight and blood glucose management strategy [82,83,84,85]. Two meta-analyses of animal models of glioma showed that KD (with or without CR) increased survival time compared to a standard diet [86,87]. Meta-analyses of publications on the impact of KD for patients with glioma showed either unestablished or beneficial outcomes for patients with glioma on KD [88,89]. Yang et al. found in a systematic review that, for cancer patients of differing types, the KD exhibited anti-tumor properties and responses only when paired with CR [90].

These findings suggest that ketogenic diets remain an area of active investigation in metabolic oncology research, although their clinical efficacy in glioma patients has not yet been clearly established [75]. Due to the potential negative impact of a high fat intake while not in keto adaptation, some clinical trials investigating ketogenic diets in glioma have monitored circulating ketone levels to assess metabolic adherence [91]. The impacts of KD on muscle wasting have been mixed and should be of concern for patients already undergoing muscle wasting and frailty, making resistance exercise a good recommendation for patients with glioma on KD [92]. Additionally, the glucose ketone index (GKI), calculated as the ratio of blood glucose to β-hydroxybutyrate (BHB), is a potential emerging biomarker for assessing the therapeutic efficacy of metabolic interventions; a lower GKI—achieved via carbohydrate or caloric restriction—previously has been associated with a reduction in glioma growth and inflammation, with improved outcomes [93].

### 4.3. Paleolithic and Whole-Food Dietary Patterns

The Paleolithic diet (paleo diet or PD) includes mostly unprocessed meats, fish, vegetables, and fruits and excludes processed foods and dairy. Despite PD not excluding carbohydrates to the level of KD, the carbohydrate sources are much lower in glycemic index than they would be in a traditional Western diet (WD) and have benefits for glycemic control and insulin signaling, though not more so than other healthy diets of low glycemic index [94]. PD has been shown to be anti-inflammatory and to aid in weight management [95,96]. PD is high in a wide variety of phytochemicals and flavonoids that may be degraded or changed in food processing in other diets. For a variety of cancers, PD was found to be inversely associated with cancer incidence and cancer-related mortality [95,97,98]. A case–control study showed PD was associated with a reduced risk of glioma incidence [99]. One popular alternative to PD is a “raw ingredient” version of PD. Heating during cooking or for sterilization/storage may negatively impact phytochemical content in the paleo diet, and an investigation into raw PD options may be an avenue for future investigations, though the current population level studies that support PD lack the resolution to separate PD and raw PD effects.

### 4.4. Mediterranean-Style Dietary Patterns

The Mediterranean diet (MD) includes a large amount of marine protein and fat sources while avoiding terrestrial sources. The MD has a much higher anti-inflammatory ω-3/ω-6 PUFA ratio compared to the typical WD and also has a lower glycemic index [100]. Though the MD was the common diet of people inhabiting Mediterranean coastal regions for centuries, it did not become popular in America until recently. MD recommendations are very similar to two other promising dietary treatments: the Dietary Approaches to Stop Hypertension (DASH), which has reduced sodium and allows refined vegetable oils in place of butter and animal fat, and the Mediterranean-DASH Intervention for Neurodegenerative Delay (MIND), which recommends particular green leafy vegetables, fruits, and berries and is associated with delayed cognitive decline [101,102]. A recent review of current case–control studies for MD, DASH, and MIND shows lowered odds ratios for incidences of glioma [74]. In contrast, a large prospective study did not, however, agree with the case–control study results and found dietary patterns similar to MD and DASH to be positively associated with glioma incidences [103]. The dietary intake was only assessed at the initial time point; therefore, adherence and dietary changes may have played a role in affecting outcomes, and the effects were not observed at a 5-year follow-up.

These dietary approaches may influence metabolic and inflammatory pathways relevant to glioma biology; however, direct clinical evidence demonstrating improved outcomes in glioma patients remains limited. Information from studies using these diets may aid in designing future glioma patient-specific diets that act at the intersection of oncology and neurology that brain cancers occupy. Along with dietary considerations, it is important to consider diet and treatment interactions. Treatment with dexamethasone increases blood glucose levels (in both diabetic and non-diabetic patients), and high cortisol increases gluconeogenesis [104,105]. This metabolic change has impacts on growth signaling for the tumor but also may impact the overall metabolic health of the patient. Considerations such as these are very important, and treatment-specific personalized diets may be an area of future research for chemical, radiological, and surgical treatment options. A summarization of the nutrition and metabolic status can be seen in Table 1.

## 5. Nutrition and the Gut Microbiota in Glioma

Interestingly, while cancer has traditionally been associated with genetic and environmental factors, recent research has revealed that the microbiota also plays a role in cancer development, progression, and response to treatment [109]. Additionally, microbial-produced metabolites may contribute to adverse outcomes in cancer treatments, as some bacteria produce metabolites which have the potential to promote oncogenesis as well as interfere with chemotherapy and immunotherapy [110,111,112]. Patients with glioma undergo treatment including surgery, radiation, chemotherapy, steroids, and antibiotics, which will cause shifts in microbial composition. This may result in a diminished microbial population, which could eliminate beneficial commensal bacteria, as well as lead to a state of dysbiosis [4,6]. Preclinical models have demonstrated that modulating the microbial composition may enhance the host’s response to cancer treatment [113,114,115,116].

### 5.1. Tumor Heterogeneity and Metabolic Context

It is important to note that gliomas are highly heterogeneous tumors, and molecular features such as IDH mutation status, MGMT promoter methylation, and tumor grade may influence metabolic activity, immune interactions, and therapeutic responsiveness [117]. For example, IDH-mutant gliomas exhibit distinct metabolic rewiring, including altered NADPH metabolism and the production of the oncometabolite 2-hydroxyglutarate, which may influence tumor–immune interactions and cellular metabolic demands. These metabolic differences may also affect how tumors respond to diet- or microbiome-mediated metabolic interventions. Therefore, additional studies investigating the microbiome or nutritional strategies in glioma will need to consider molecular subtype and tumor metabolic phenotype, as these factors may influence treatment responsiveness and therapeutic outcomes [118].

### 5.2. Diet and Microbial Composition

Poor diets are often newer-age diets, such as the WD, and include quantities of convenience foods, refined grains, processed meats, sugary foods and beverages, candies, fried food, conventionally farmed animal products, high-fat dairy, and foods rich in high-fructose ingredients [119]. In terms of macronutrients, this results in a 43% carbohydrate, 40% fat, and 17% protein macronutrient ratio, which is suboptimal, as such dietary patterns have been associated with metabolic disease and microbial dysbiosis [120,121]. The WD has been associated with the development of metabolic diseases, obesity, hypertension, and cancer [122]. Additionally, the WD has been linked to increased levels of endotoxin-producing bacteria, leading to inflammation, dysbiosis, and, in severe cases, endotoxemia (elevation of plasma levels of lipopolysaccharides) [123,124]. Filippis et al. found that a strict adherence to a MD resulted in beneficial microbes with associated metabolites [125]. The advantages of this diet may come from foods such as fruits, vegetables, and legumes, as these are considered prebiotic fibers which serve as an energy source for the gut microbiota and support preferable microbes, as well as the production of metabolites.

The MD has an abundance of polyphenols and omega-3 fatty acids, which have been noted to modify the microbial composition [126]. Costantini et al. [127] observed a reduced abundance of *Faecalibacterium*, along with increased Bacteroidetes and butyrate-producing bacteria from the Lachnospiraceae family, via the MD. The current literature does not have a published optimized diet to improve microbial composition; however, dietary intervention does incur rapid changes in microbial composition within 24 to 48 h [128]. It is important to note that the microbial composition will return to the previous composition if the new diet is not continued [129].Therefore, dietary intervention for beneficial microbial changes must be adhered to in the long term.

### 5.3. Probiotics and Prebiotics in Cancer Care

The current hypotheses regarding the use of probiotics extend beyond modulating the microbiota; they also suggest potential benefits for systemic immune function and metabolic responses. Post-surgical patients who receive probiotics may benefit from a reduction in systemic infections, improved bowel function, less postoperative pain, lowered risk of antibiotic-related diarrhea and decreased incidence of anastomotic leaks [130]. There has been progression in perioperative management with ERAS protocols; however, bacterial infections remain a major challenge [131]. Surgery and related medications can induce inflammation and suppress the immune response, potentially weakening the gut mucosal barrier. This may result in alterations to the microbial composition, leading to the loss of beneficial bacteria and an overgrowth of pathogenic or harmful microbes [132]. Probiotics and prebiotics have been shown to support, restore and modulate the microbial composition during this time, which reduces the risk of dysbiosis and infection [133,134]. A meta-analysis supported that the usage of probiotics had a reduction in complications like diarrhea, pneumonia, sepsis, and urinary tract infections and a positive impact on the duration of antibiotic treatment, postoperative fever, and length of hospital stay. Tang et al. conducted a meta-analysis which included 1776 participants and compared probiotic and synbiotic supplementation against a control. They found that probiotic and synbiotic supplementation improved gut motility [135]. Additional randomized controlled trials provide further support for the use of probiotics, prebiotics, and synbiotics in postoperative patients [136,137,138,139].

### 5.4. Microbial Taxa Associated with Treatment Response

*Akkermansia muciniphila* is a Gram-negative bacterium, typically colonizing the intestinal tract in healthy adults, that adheres to the mucosal layer in the intestine. *A. muciniphila* has been associated with successful immunotherapy treatment in several cancers [140]. Routy et al. found that the gut microbiota influenced the efficacy of anti-PD-1-based immunotherapy against epithelial tumors. They demonstrated that antibiotics inhibited the clinical benefits of anti-PD-1 treatment in patients with advanced cancer and used fecal microbiome transplants in gnotobiotic mice to confirm the role of the microbiota in anti-PD-1 response. Mice with a microbiome from a responsive patient responded to anti-PD-1 treatment, and mice with a non-responder microbiome did not. The responder microbiome resulted in a large abundance of *A. muciniphila*, and, when *A. muciniphila* was orally supplemented, this restored the effect of anti-PD-1 treatment in the non-responders [141]. Likewise, a large meta-analysis was conducted in 338 non-small-cell lung cancer patients, in which a larger abundance of *A. muciniphila* was associated with a more inflamed tumor microenvironment, inferring that there is a response to anti-PD-1 treatment [140]. The mechanism behind the beneficial effects of *A. muciniphila* is still largely unknown; however, Zhu et al. tested live *A. muciniphila*, pasteurized *A. muciniphila*, and the Amuc_1100 protein and discovered that *A. muciniphila* in its live form effectively stimulates MHC-II-plasmacytoid DC pathways while reducing CXCL3 expression within Biglycan (Bgn)- and Decorin (Dcn)-expressing cancer-associated fibroblasts (CAFs). Mechanistic studies suggest that *A. muciniphila* may influence antigen presentation and T cell activation pathways, although these mechanisms remain under investigation [142]. *A. muciniphila* was reduced in abundance in mice by 66% whilst on a high-fat WD; however, the supplementation of *A. muciniphila* reversed these changes [143]. Interestingly, studies have also found that a diet low in fermentable carbohydrates, known as a FODMAP diet (Fermentable Oligo-, Di-, Monosaccharides, and Polyols), can promote an increase in *A. muciniphila* abundance [144].

Additionally, *Lactobacillus planatrum* and *Bifidobacterium lactis* have been deemed common beneficial strains in the model of glioma. Wang et al. discovered that the combination of *L. plantarum* and *B. lactis* reduced tumor volume, prolonged survival, and repaired intestinal damage in a glioma mouse model [145]. The potential mechanism behind the benefit of *L. planatrum* and *B. lactis* may be due to their role in the downregulation of the PI3K/AKT pathway (a key pathway in cancers, as it influences overall cell growth, metabolism, and survival). For example, *L. plantarum* supplementation led to a notable decrease in *Staphylococcus* and *Heliobacter* [146]. Supporting beneficial bacteria via the implementation of probiotics is essential to attempt to colonize supportive microbes; however, probiotics get significantly reduced via the oral passage through the gastrointestinal tract. Cook et al. showed that other *Bifidobacteria* members, *B. longum* and *B. breve*, were undetectable after passing through gastric juice, and this is not including the passage through the small intestine (bile acid and enzymes) and colonization resistance [147,148]. *L. planatrum* and *B. lactis* can be found in fermented foods, and inulin has been shown to increase the overall abundance of *L. planatrum* [149]. Likewise, *B. lactis* cannot break down all sources of fiber, but does contain an α-l-arabinofuranosidase, which enables the breakdown of arabinoxylan, which is found in the cell walls of many plants and grain-based foods [150]. This indicates that specific prebiotics may be necessary to promote the growth and colonization of selective bacteria [151].

### 5.5. Microbial Metabolites and Immune Modulation

Mice with a depleted microbiome have a reduced response to ICB therapy (CTLA-4 blockade); however, specific bacteria have restored the response to immunotherapy, such as *Bacteroides fragilis* and *Bacteroides thetaiotaomicron*. Additionally, Dees et al. utilized a novel humanized mouse microbiome model, where mice were transplanted with microbiota from multiple healthy human subjects. These, humanized microbiome mice were given GL261 gliomas and treated with anti-PD-1 therapy, and responders to treatment had a larger abundance of *Bacteroides cellulosilyticus* [115]. Members of the *Bacteroides* genus are key producers of SCFAs in the gut, primarily generating propionate, butyrate, and acetate. In preclinical models, reduced levels of SCFAs—alongside decreases in histamine, adenosine, norepinephrine, 5-hydroxyindoleacetic acid (5-HIAA), GABA, and tryptophan—were associated with changes in immune function. In patients with glioma, lower fecal levels of metabolites like 5-HIAA and norepinephrine were also observed. Notably, SCFAs such as acetate, butyrate, and propionate were diminished in late-stage GL261 mouse models of glioma, suggesting a potential impact on the tumor microenvironment [152]. The loss of SCFAs may affect glioma-related immune responses, influencing critical cells such as macrophages, microglia, and T cells. SCFAs are known to support regulatory T cells, and butyrate is essential for the effector functions of cytotoxic lymphocytes, contributing to antiviral and anti-tumor immunity [152].

**Figure 1:**
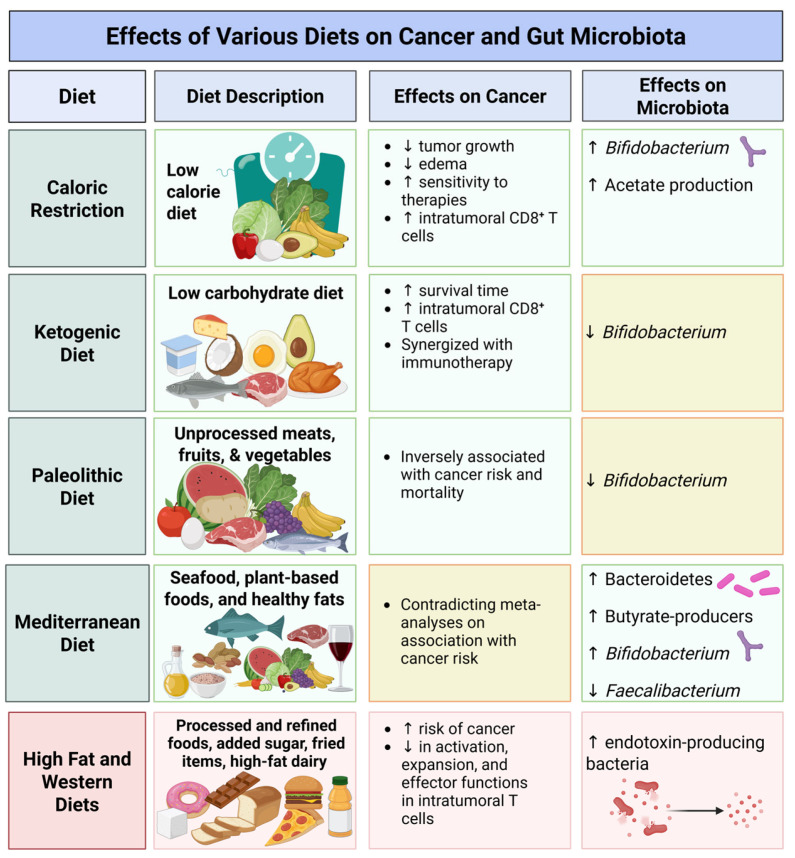
A summarization of the effect of diet on all cancers and the microbiota. Created in BioRender. Cox-Holmes, A. (2025).

*Bifidobacterium* is another genus that has been positively associated with anti-tumor response and immunotherapy efficacy. For example, Matson et al. found *Bifidobacterium longum* to be more prevalent in patients with melanoma who responded to anti-PD-1 blockade, and Zhao et al. found *Bifidobacterium breve* to be a biomarker for anti-PD1 response in Chinese patients with non-small-cell lung carcinoma [153,154]. Similarly, one study found that the oral administration of *Bifidobacterium* alone was as effective as anti-PD-1 in improving anti-tumor response in mouse models of melanoma [155]. While the exact mechanisms of the role of *Bifidobacterium* in anti-tumor response are still being investigated, *Bifidobacterium* has been shown to promote Th1 polarization, improve dendritic cell activity, and enhance CD8^+^ T cell activation [156]. Specific dietary components such as fiber, polyphenols, and flavonoids have all been associated with an increase in *Bifidobacterium* [157]. The MD, which is rich in fruits, vegetables, and legumes containing these compounds, has been similarly linked to a higher *Bifidobacterium* abundance [158,159,160]. In contrast, low-carbohydrate diets like the Paleolithic and ketogenic diets tend to decrease *Bifidobacterium* levels [161,162,163,164]. This decrease may be attributed to the reduced fiber intake in these diets. Additionally, one study found that the ketone body BHB, produced during a KD, selectively inhibited *Bifidobacterium* growth [165]. Thus, while the KD holds promise for anti-tumor support, its effects may involve mechanisms independent of *Bifidobacterium*. A summarization of the effect of diet on cancer and the microbiota can be observed in Figure 1.

Overall, emerging evidence suggests that diet–microbiome interactions may influence immune signaling and therapeutic responses in cancer. However, most findings relevant to glioma are derived from preclinical models or extrapolated from other malignancies. Consequently, the role of microbiome-targeted nutritional strategies in glioma management remains an area of active research rather than an established clinical intervention.

## 6. Nutritional Support for Perioperative Recovery

### 6.1. Current Perioperative Nutritional Guidelines and Screening

Surgical resection is required for most patients with glioma to lower intracranial pressure and reduce tumor burden [166,167]. Current perioperative guidelines have limited information on both pre- and postoperative nutritional requirements, the lack of which could result in increased incidences of cancer-related malnutrition [168]. Perioperative fasting, which requires patients to fast from food and drinks the night prior to surgery, was originally implemented to prevent aspiration and postoperative ileus. However, increasing evidence suggests that liquid nutrient supplementation is safe up to two hours prior to surgery [169,170]. The European Society for Clinical Nutrition and Metabolism (ESPEN) has developed Enhanced Recovery After Surgery (ERAS) guidelines to advocate for perioperative nutrition reform [171]. ERAS’s suggested nutritional reforms include preoperative digestible carbohydrate supplementation via liquid nutritional supplements consumed up to two hours prior to surgery, as higher-carbohydrate diets have been shown to decrease hospital stays by 1.5 days and improve insulin resistance postoperatively [170,172]. ERAS also advocates for an earlier postoperative dietary intake, when possible, to help decrease the metabolic deficit caused by fasting prior to surgery and the increased metabolic demands throughout the wound healing process after surgery [173]. These novel ERAS nutritional guidelines have been adopted via multiple hospital systems and should be considered for resection surgery.

Prior to surgery, it is first recommended to determine the nutritional status of cancer patients via assessments like the Malnutrition Universal Screening Tool or the Nutritional Risk Screening-2002 [174,175]. The catabolic nature of cancer commonly results in malnutrition, which may increase postoperative mortality and prolonged hospital stays [168,176]. Huq et al. evaluated which nutritional assessment best predicted postoperative survival within GBM patients using the albumin/globulin ratio (AGR), nutritional risk index (NRI), and prognostic nutritional index (PNI) [177]. Their study of 242 patients with a mean age of 57.6 years suggested that PNI (determined via serum albumin levels and absolute lymphocyte counts) was the best predictor of postoperative survival, with a clinically actionable cut point of 43.38. The PNI is a calculated metric that divides patients into high-risk (≥43.8) and lower-risk (<43.8). AGR did not have any predictive value on postoperative survival, while NRI (determined via albumin levels and weight loss) and albumin levels alone still had predictive value; however, they did not perform as well as PNI [177].

### 6.2. Preoperative Carbohydrate Loading Versus Carbohydrate Restriction

Pang et al. analyzed 26 studies to determine the efficacy of ERAS protocols (increased carbohydrate loading) for surgical oncology patients. Carbohydrate loading resulted in better short-term outcomes for cancer surgeries [178]. Pang et al. noted that, while ERAS improved postoperative recovery, the effect on metastasis or recurrence needs to be further investigated [178]. Re-evaluating the type and quality of carbohydrate supplementation administered may mitigate the risks of increased malignancy caused via carbohydrate loading [179,180]. Alternative carbohydrate options that reduce glycemia may provide a replacement for current carbohydrate supplements [179,180].

ERAS protocols recommend short-term preoperative carbohydrate loading to reduce surgical stress and support postoperative recovery, whereas carbohydrate-restricted strategies such as ketogenic diets have been proposed as longer-term metabolic interventions intended to influence tumor metabolism. These approaches occur at different clinical stages and serve distinct physiological purposes. Clinically, this suggests that nutritional priorities may differ across glioma management, with perioperative care focused on recovery and later treatment phases potentially emphasizing metabolic modulation. Therefore, ERAS carbohydrate loading and longer-term carbohydrate restriction should not necessarily be viewed as contradictory, but rather as phase-specific strategies that require further clinical evaluation in glioma patients [171].

ERAS-recommended preoperative carbohydrate supplements contain grain-derived maltodextrin as the carbohydrate source, with a potential inclusion of fructose in some formulations. Maltodextrins are glucose polymers that are rapidly absorbed within the small intestine, leading to higher blood glucose levels [181,182]. The beneficial effects of preoperative carbohydrate supplements need to be weighed against the increased malignancy associated with elevated blood glucose [179,180,183,184]. One way to mitigate hyperglycemia is to consume carbohydrate sources that are rich in fiber or soluble fiber, which require microbial activity for absorption, thereby delaying digestion [185,186]. One such carbohydrate that has been studied is resistant maltodextrin (RM), which requires microbial fermentation [187]. This reliance on microbial fermentation reduces the percentage of RM that is absorbed within the small intestine before colonic fermentation converts more of the RM molecule into a bioavailable energy source [187]. Other prebiotics that have been used in supplementation and could be considered for nutritional interventions in patients with gliomas include inulin, fructo-oligosaccharides, galacto-oligosaccharides, and fructans [188,189]. Ensuring that fiber-rich carbohydrate sources are consumed prior to surgery may help to mitigate hyperglycemic states, while also providing prebiotics for the gut microbial production of short-chain fatty acids (SCFAs), a group of microbial metabolites known to have a wide range of beneficial effects on the host [187].

### 6.3. The Role of Pre-, Post- and Synbiotics in Perioperative Care

Probiotics, which are viable microbial strains, are also important in promoting positive perioperative outcomes. Zaharuddin et al. supplemented strains of *Lactobacillus* and *Bifidobacterium* for six months in colorectal cancer patients beginning at four weeks after surgery and observed decreased inflammatory cytokines in the probiotic-supplemented patients [190]. Additionally, synbiotics are foods that combine the benefits of both pre- and probiotics like fermented milk products (kefir or yogurt) or fermented vegetables (e.g., sauerkraut). The combined effect results in significantly improved outcomes compared with either prebiotics or probiotics alone, suggesting that synbiotics should be considered in the perioperative nutritional regimen [191,192]. More work is needed to recommend consumption rates for different synbiotics in patients with glioma in either the preoperative or postoperative state.

### 6.4. Protein and Immunonutrition in Recovery

Increased perioperative dietary protein is essential on a cellular level for remodeling a large portion of the injured tissue after surgery. Most research focuses on the ability of dietary protein to prevent muscle catabolism in a negative nitrogen balance state, as sarcopenia has been associated with poor prognoses [173,193,194,195]. The current recommendations for protein consumption suggest 1.2 g to 2.0 g protein per kilogram of body weight per day, with sources that contain a high essential amino acid (EAA) content and bioavailability and a high ileal digestibility [195]. In addition to selecting protein sources with sufficient EAA profiles like animal proteins, whey or eggs, Rittig et al. observed a 20% decrease in whole-body protein catabolism in human males given additional EAA supplementation [196]. The role of increased protein intake requires further evaluation.

ESPEN and ERAS have recommendations on oral nutritional supplementation for surgical oncology patients that include immunonutrition [197]. Immunonutrition is the supplementation of nutrients that have exhibited positive modulatory effects on the immune system, normally including arginine, ω-3 fatty acids, nucleotides, and, in certain cases, glutamine [198,199]. A meta-analysis by Yu et al. supported the use of postoperative nutritional supplementation, showing that it reduces hospitalization time in patients and decreases the risk of surgical wound infections [199]. Further studies are needed to better understand the role of immunonutrition in the treatment of gliomas specifically.

### 6.5. Micronutrients in Postoperative Recovery

Vitamin C, which is water soluble, is suggested to increase benefits after musculoskeletal injuries since it has been shown to increase collagen synthesis and modulate oxidative stress [200,201]. Patients who received vitamin C supplementation after surgery showed decreased inflammatory markers, such as the erythrocyte sedimentation rate (ESR) and C-reactive protein (CRP), and experienced decreased hospital stays [202,203].

Vitamin D, an important fat-soluble vitamin, has also been identified in musculoskeletal injuries for its ability to affect calcium and phosphate levels, while also preventing muscle atrophy [204]. Additionally, Wu et al. noted a dysregulation of the inflammatory response after cutaneous injury in mice with vitamin D deficiency [205]. Within the nervous system, vitamin D has been associated with axonal survival, myelination, regeneration, and diameter increase, as well as neuronal survival, with vitamin D3 being more potent than D2 [206,207,208,209,210].

A subset of B vitamins is called neurotropic B vitamins (vitamin B1, B6, and B12), which have been extensively studied for their role in regeneration and environmental protection within nervous tissue [211]. Additionally, magnesium is a cofactor and has been associated with lowered inflammation and, for this reason, could potentially improve postoperative outcomes [212,213]. Magnesium has a wide scope of effects ranging from antagonistic effects to calcium-dependent apoptosis to promoting Schwann cell proliferation and axon regeneration [213,214,215,216,217,218]. Until a better understanding of the effects of magnesium supplementation can be determined, it should only be considered in patients with hypomagnesemia.

However, most studies evaluating micronutrient supplementation have been conducted in broader surgical or musculoskeletal contexts, and evidence specific to glioma perioperative care remains limited.

## 7. Nutrition, Metabolism, and the Anti-Tumor Immune Response

### 7.1. Metabolic and Immunological Features of the GBM Tumor Microenvironment

GBM has a highly immunosuppressive tumor microenvironment (TME). The tumor recruits immune cells, such as tumor-associated macrophages (TAMs), which make up 30% of tumor mass, and reprograms them to an immunosuppressive phenotype [219,220]. GBM cells secrete epidermal growth factor (EGF) and transforming growth factor-β (TGF-β), which not only stimulate tumor growth but also suppress the anti-tumor immune response [221]. The tumor cells and endothelial cells also aid in immune evasion by increasing the surface expression of programmed death-ligand 1 (PD-L1), which further inhibits T cells [222]. The main immunosuppressive immune cells in the TME of GBM consist of M2 TAMs and microglia as well as myeloid-derived suppressor cells (MDSCs), tumor-associated neutrophils (TANs), and regulatory T cells (Tregs) [223]. TAMs produce anti-inflammatory cytokines such as interluekin-10 (IL-10) and TGFβ, as well as angiogenesis- and tumor-promoting factors such as vascular endothelial growth factor (VEGF), EGF, and platelet-derived growth factor (PDGF), and upregulate PD-L1 [224]. MDSCs can be either granulocytic or monocytic, and monocytic MDSCs are more common in the GBM TME [225]. These MDSCs aid in immune evasion by upregulating arginase 1 (Arg1) production, activating Tregs, and inducing T cell apoptosis [226]. Tregs also regulate effector T cells through IL-10 production and the upregulation of the immunosuppressive receptors cytotoxic T-lymphocyte-associated protein 4 (CTLA-4) and programmed death-1 (PD-1) [227].

GBM also promotes immunosuppression through metabolic reprogramming, producing lactic acid and creating an acidic TME that fuels tumor growth while promoting immune cell apoptosis [228]. Additionally, the activation of the PI3K/Akt/mTOR pathway enhances glycolysis and anabolic processes, supporting tumor proliferation and further suppressing immune responses by impairing the function and viability of anti-tumor immune cells [229]. Some GBM cells also exhibit “glutamine addiction,” meaning they are dependent on glutamine metabolism as a carbon and nitrogen source [230]. Beyond its anabolic role, glutamine can also be fermented for energy production via mitochondrial substrate-level phosphorylation [93]. The overexpression of c-Myc is common in GBM cells and has been shown to increase the activity of glutamine amidohydrolase and enhance the uptake of glutamine through the ASCT2 transporter [231,232]. This glutamine dependency can lead to its depletion in the TME, impairing immune cells that also rely on glutamine for function [219]. Importantly, the acidification of the TME is driven not only by lactate production from glycolysis but also by succinate accumulation from glutaminolysis, both of which contribute to immunosuppression. GBM cells also increase lipid accumulation and synthesis, and as a result lipid mediators like prostaglandin E2 (PGE2) accumulate in the TME [233,234]. PGE2 accumulation can lead to immunosuppressive effects such as limiting the expansion of tumor-infiltrating effector T cells and M2 polarization [235,236].

As metabolism is altered by the GBM tumor, dietary modulation has been proposed to improve tumor response to treatment. One of the important bridges between diet and anti-tumor immune response is the gut microbiota. However, the presence of the tumor itself has been observed to modulate the gut microbiota composition, and the therapies used to treat GBM are also known to impact the gut microbial composition [237].

### 7.2. Nutrients and Immune Cell Function

Before understanding how diet and nutrition impact the immune response to cancer, it is important to understand that many dietary nutrients directly play a role in immune development and maintenance. Amino acids are important regulators of both innate and adaptive immune cells. Arginine is a substrate of nitric oxide synthase and can contribute to the production of nitric oxide, a cytotoxic compound secreted by M1 macrophages [238]. The arginine degradation pathway has also been linked to NF-κB, one of the major inflammatory signaling pathways [239]. Tryptophan is essential for immune cell division and development, as it is necessary for protein synthesis [240]. Tryptophan degradation also results in NAD+, which can initiate programmed cell death in T cells [241]. Many vitamins are also immune-regulatory. Notably, vitamin A promotes FoxP3^+^ Tregs and Th2 cells while blocking Th1 and Th17 cells [242,243,244,245]. Deficiencies in vitamin A also led to a suppression of NK cells and lessened phagocytosis for macrophages [246]. Vitamin D is also known to inhibit pro-inflammatory Th1, Th9, Th22, and Th17 cells while promoting Th2 and Treg cytokines [247,248]. It has also been shown to promote phagocytosis and bacterial killing in myeloid cells [249]. Vitamin C has been shown to enhance phagocytosis and ROS generation by neutrophils and macrophages and enhances the differentiation and proliferation of B and T cells [250,251,252,253,254,255].

Various lipids can be either pro-inflammatory or anti-inflammatory. Saturated fatty acids (such as palmitic acid, stearic acid and lauric acid) as well as sterols (cholesterol) are primarily known to be pro-inflammatory [256]. Palmitic acid treatment in macrophages increased chemokine and cytokine expression and reduced phagocytic capacity [257,258,259]. Palmate is also a toll-like receptor 4 (TLR4) ligand and induces NF-κB and inflammasome activation in macrophages and dendritic cells (DCs) [260,261]. Feeding mice a diet rich in saturated fatty acids led to an increase in the inflammatory profile in naïve T cells but an impairment in the activation and proliferation of antigen-experienced T cells [262], suggesting that, while high-fat diets lead to low-grade systemic inflammation, they may also weaken defense against infections and cancers. High-cholesterol diets have been shown to increase mast cell (cells producing histamine and heparin, typically associated with allergic inflammation) activation in mice [263]. These findings are largely derived from animal studies and require validation in humans. Unsaturated fatty acids, however, can be either pro- or anti-inflammatory. Omega-3 fatty acids like eicosapentaenoic acid, docosahexaenoic acid, and ω-3 PUFAs have been found to decrease circulating inflammatory cytokines and promote an anti-inflammatory M2 macrophage phenotype [264,265,266]. Prostaglandins, such as PGE2, can be derived from polyunsaturated fatty acids and have an anti-inflammatory role in chronic inflammation, but a pro-inflammatory role in acute inflammation [267,268].

Minerals, such as zinc (Zn) and selenium (Se), are metals found in shellfish, plants, and other foods that are essential for biological functions and impact the immune system. Zinc, or the ion Zn^2+^, is involved in the proliferation and differentiation of T cells. Zn^2+^ signals are necessary in the induction of Th1 differentiation and lead to the upregulation of CD69, IRF-1 and KLF10 [269]. Interestingly, zinc supplementation has also been shown to induce Treg differentiation and activation, while also limiting inflammatory Th1 and Th2 numbers in elderly patients [270,271,272]. Supporting the anti-inflammatory role, zinc deficiencies have been associated with increased inflammation, and high cytokine levels have been associated with low plasma zinc levels [273]. Another study showed that CD4 coupling with Lck, which initiates the T cell co-receptor signal, was dependent on the Zn^2+^ concentration [274]. Selenium has also been shown to promote Th1 differentiation in mice [275]. Furthermore, selenium supplementation in patients with childhood cancer increased IgG and IgA as well as neutrophil numbers [276].

### 7.3. Dietary Influences on Anti-Tumor Immunity

Many popular dietary interventions discussed earlier can also influence the immune response to cancers including GBM. Some diets, such as CR, KD, high-fiber diets, and low-protein diets, may be beneficial to the anti-tumor immune response, while others like the WD and high-fat diet (HFD) may be detrimental to the anti-tumor immune response [277,278,279]. Dietary supplementations have also been shown to modulate the immune response to tumors [75].

CR and fasting have been shown to improve the anti-tumor immune response in various types of cancer. Mao et al. showed that CR improved the anti-tumor IFNγ^+^ CD8^+^ T cell response to MC38 colon adenocarcinomas [280]. In mouse and human breast cancer, CR with radiotherapy increased CD8^+^ T cells and decreased Tregs in the tumor microenvironment [281]. A separate study in mouse models of breast cancer showed that fasting decreased heme oxygenase-1 in the tumor cells, and when it was combined with doxorubicin (chemotherapy) there was an increase in common lymphoid progenitor cells in the bone marrow, an increase in the recruitment of CD8^+^ T cells to the tumors, and reduced Tregs [282]. In a Lewis lung carcinoma model, fasting reduced insulin-like growth factor 1 (IGF-1) levels, which sensitized tumors to anti-PD-1 therapy, leading to an increase in intratumoral CD8^+^ T effector cells and a downregulation of exhaustion markers on T cells [283]. Interestingly, researchers have also shown that fasting reduced insulin-like growth factor 1 (IGF-1) levels and sensitized mouse and rat glioma cells to TMZ and cyclophosphamide chemotherapies, as well as radiotherapy [78,284].

Similar to CR, low-protein diets have been shown to increase immune cell recruitment to the tumor. In mouse models of lymphoma (Eμ-Myc), colon cancer (CT26), and melanoma (B16), feeding mice isocaloric diets with 25% less protein increased survival by inducing IRE1a ER stress and RIG1 damage-associated pattern recognition pathways [285]. This led to an increase in intratumoral CD8^+^ T cells and natural killer (NK) cells. Because IRE1a signaling has also been shown to increase CD8^+^ and perforin expression in GBM, a similar diet may also be beneficial for patients with GBM [285]. A low protein intake has also been associated with lower levels of IGF-1 and improved cancer survival [286], so it may modulate the immune response similar to the mechanisms discussed for CR diets. Orillion et al. found that dietary protein or amino acid (methionine or cysteine) restriction inhibited the polarization of macrophages to a pro-tumor M2 phenotype and improved CD8^+^ T cell infiltration and response to mouse models of prostate (RP-B6-Myc) and kidney (RENCA) cancer [287]. In mouse melanoma models B16F10 and MC38, the dietary restriction of methionine improved the response to radiation and immunotherapy by increasing the infiltration of T cells and DCs in the tumor microenvironment [288]. This is because the restriction of methionine increases cGAS activity by preventing methylation. The double deprivation of methionine and cysteine led to triggered autophagy and increased ROS, and methionine restriction improved the efficacy of TMZ chemotherapy in U87 glioma models (but this was immune-independent, as it was performed in nude mice) [12,289]. However, seeing as T cells also require methionine for proliferation and survival, methionine restriction may not be beneficial to the anti-tumor immune response [290].

The KD has also been noted to modulate T cell migration and function. Mice on a KD had an improved response to CT26 colon tumors due to an upregulation in chemokines, which recruited neutrophils, NK cells and effector T cells. These T cells were also shown to have an increase in IL-2 and IFNγ production [291]. This was also seen in the GL261 glioma model, where mice fed a KD had an increase in CD4^+^ T cells and IFNγ^+^ CD8^+^ T cells, with no change in Treg numbers and a reduction in immunosuppressive receptors and ligands including PD-1, CTLA4, and PD-L1 [292]. However, a 2022 paper by Kesarwani et al. [293] found that, while a KD elicits anti-tumor activity, it paradoxically increased M2 (pro-tumor) macrophages. The anti-tumor response and survival were further improved when KD was combined with a CSF-1 inhibitor [293]. Other studies have also shown that KD synergizes with anti-PD-1 and anti-CTLA-4 blockade to improve the anti-cancer immune response [294,295,296]. Seeing as the KD alone has shown benefits in mouse models of glioma and it synergizes with ICB in other models, it may represent a potential strategy worth further investigation in GBM.

Many studies have shown that high-fiber diets can improve the anti-tumor immune environment in various cancers through the gut microbiota–immune axis. Mice with bladder tumors (UPPL1591) that were fed modified diets high in fiber (with psyllium and inulin) had an increase in survival as well as an increase in CD8^+^ T cell infiltration into the tumor [297]. Supplementation with inulin and mucin fibers has been shown to increase the infiltration of effector (CD44^hi^) T cells, IFNγ^+^ CD4^+^ T cells, and DCs into the tumors of melanoma-bearing mice, leading to an increase in survival [298]. Although direct studies in GBM are lacking, dietary fiber may influence anti-tumor immunity through microbiome-derived metabolites, as human gut microbiota have been shown to affect glioma immunotherapy responses in preclinical models [115].

A recent study by Kim et al. showed that supplementation with a high-glucose drink (HGD) increased the anti-tumor immune response in preclinical GBM (GL261- and CT-2A-bearing mice) and improved anti-PD-1 efficacy in these mice [299]. Unlike high-sugar diets, this short-term high-glucose treatment showed no evidence of metabolic disease and was beneficial. A single-cell analysis of the TME showed that HGD increased the CD8^+^ T cell/Treg ratio as well as the CD4^+^ T cell/Treg ratio and was associated with an increase in interferon-related genes (*fi27l2a*, *Ifit1*, *Ifit2,* and *Ifit3*). Similar to the aforementioned fiber supplementation studies, the induction of an anti-tumor response from the HGD was dependent on the gut microbiota, and the treatment of GF glioma-bearing mice with *Desulfovibrio* bacteria and HGD similarly improved the survival and frequency of cytotoxic CD8^+^ T cells and a population of cytotoxic CD107a^+^NKG2D^+^CD4^+^ T cells [299]. Interestingly, this paper also found that probiotics alone did not increase anti-PD-1 efficacy in the mice transplanted with glioma.

**Figure 2:**
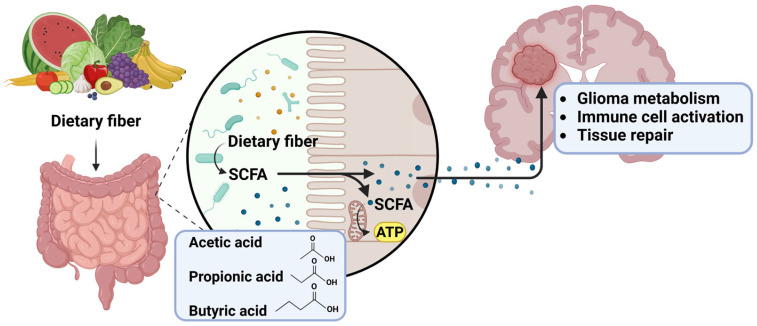
The beneficial effects of dietary fiber, highlighting how gut microbiota ferment fiber to produce short-chain fatty acids, which exhibit anti-tumor properties in glioma. Created in BioRender. Cox-Holmes, A. (2025).

SCFAs are produced by the gut microbiota as the microbes break down dietary carbohydrates. The three SCFA compounds produced are butyrate, propionate, and acetate. Butyrate has been shown to have anti-tumor effects [300]. Additionally, butyrate has been observed to promote the differentiation of monocytes into macrophages. Zhou et al. (2024) [301] corroborated that gut dysbiosis promoted tumor growth and expanded the presence of M2-like macrophages within the TME, coinciding with a decrease in the overall presence of SCFAs. Introducing SCFAs resulted in shifting the macrophage population towards an M1-like phenotype and contributing to improved glioma outcomes [301]. Butyrate improves the efficacy of anti-PD-1 therapy by stimulating the activation of CD8^+^ T cells, resulting in improved outcomes [302,303]. Additionally, Zhu et al. supported that butyrate supplementation promoted anti-PD-1 anti-tumor efficacy via the modulation of T cell receptor signaling on cytotoxic T cells [303]. Propionate contributes to immune modulation via interleukins, cytokines, and oxidation stress, while also inhibiting NF-κB and histone deacetylase pathways and upregulating PPAR-γ (peroxisome proliferator-activated receptor gamma, which is a nuclear receptor crucial for controlling gene expression associated with metabolism, inflammation, and cell differentiation) [304]. Acetate can be utilized as an energy source for GBM or brain metastases. Wang et al. revealed that supplementing acetate supported tumor growth and reduced overall CD8^+^ T cell infiltration into the TME, and blocking acetate uptake hinders immune evasion, thereby boosting the effectiveness of anti-PD-1 therapy [305]. However, when CD8^+^ T cells are activated by IL-12, they experience a rise in intracellular acetyl CoA levels, enabling them to continue producing IFNγ even in nutrient-depleted, tumor-conditioned media. This suggests that CD8^+^ T cells may need a certain acetate level for activation, which could enhance anti-tumor activity [306] (Figure 2). Overall, these findings suggest that microbiome-derived SCFAs may influence tumor immunity; however, most evidence derives from preclinical models, and further studies are needed to determine their relevance in glioma patients.

However, not all diets are beneficial. The WD or HFD are examples of diets that are detrimental to the anti-tumor immune response. For example, the WD has been linked to an increased risk of colon cancer [307]. A possible explanation of this risk is an increase in colonic inflammation on a WD. In a mouse model of sporadic colon cancer, a WD led to an increase in oxidative stress, shown by a decrease in endogenous methionine, a decrease in the ratio of cysteine to cystine, and an increase in the expression of inflammatory proteins such as IgA, C-reactive protein, MPO, and MIP-1 [308]. In multiple mouse cancer models, HFD-induced obesity led to a decrease in CD8^+^ T cells in the TME, and fewer of these CD8^+^ cells were activated (ICOS^+^ or CD44^+^), expanding (Ki67^+^), or effectors (GZMB^+^) [309]. Mechanistically, they found that an HFD increases serum free fatty acids (FFAs), but the tumor cells are metabolically reprogrammed by this and deplete the FFAs in the TME, leaving limited fuels for the T cells to use [309]. Xu et al. [310] found that oxidized low-density lipoproteins (OxLDLs) accumulate in the TME in murine B16 and MC38 tumors, and that exhausted (PD-1^+^ Tim3^+^) CD8^+^ T cells had higher amounts of LDL and cholesterol than other tumor-infiltrating lymphocytes [309]. They found that CD36 on these T cells leads to the uptake of OxLDLs, and this induces oxidative stress programs like lipid peroxidation, which result in p38 MAPK phosphorylation and the inhibition of T cell effector functions [309]. Fatty acid metabolism has also been shown to impact immune cells in the TME of GBM. In wild-type and radioresistant U251 and GL261 glioma models, fatty acid oxidation led to an increase in CD47 (a “don’t eat me” signal) expression on tumor cells, which reduced tumor phagocytosis by macrophages [311].

## 8. Conclusions

Nutrition represents an important component of supportive care for patients with glioma and may influence treatment tolerance, metabolic status, and immune function. Emerging research suggests that diet–microbiome interactions could play a role in modulating the tumor microenvironment and therapeutic responses. However, much of the current evidence derives from preclinical models or observational studies, and well-designed clinical trials are needed to determine the translational relevance of specific dietary strategies. Future work integrating nutritional science, tumor biology, and microbiome research may help identify personalized dietary approaches that improve patient quality of life and clinical outcomes.

## 9. Limitations

Several limitations should be considered when interpreting the literature summarized in this review. First, much of the available evidence is derived from preclinical studies, including murine glioma models and in vitro systems, which may not fully recapitulate the complexity of human disease. Additionally, substantial heterogeneity exists in study design, including differences in dietary assessments, microbiome sequencing methodologies, patient treatment regimens, and tumor molecular subtypes. These factors make cross-study comparisons challenging. Furthermore, the gut microbiome is influenced by numerous confounding variables such as antibiotic use, corticosteroid treatment, chemotherapy, and lifestyle factors, which are common in glioma patient populations and may complicate the interpretation of microbiome–diet interactions.

## 10. Methods of Review

This manuscript was conducted as a narrative review. The relevant literature was identified through searches of major biomedical databases using combinations of terms including glioma, nutrition, metabolomics, diet and cancer, microbiota, and the gut–brain axis. Additional articles were identified through reference lists of relevant publications and the authors’ subject matter expertise. Given the narrative nature of the review and the involvement of multiple contributing authors, a standardized systematic search strategy was not applied; instead, the goal was to synthesize representative and relevant studies that illustrate the current knowledge regarding the interaction between nutrition, the microbiome, and glioma.

## Figures and Tables

**Table 1:** A summary table of the nutritional studies and summarized results.

Nutrient/Metabolic Status	Study Type	Outcomes	Reference
Hyperglycemia	Meta-Analysis of Glioma Patient Studies	Reduced survival time	[36]
Hyperglycemia	Meta-Analysis of Glioma Patient Studies	Reduced survival time	[37]
PUFA Intake	Case–Control Study	PUFA intake in the highest quartile lowered glioma risk	[58]
Dietary Inflammatory Score	Case–Control Study	Higher inflammatory diet score increased glioma risk	[59]
Antioxidants	Meta-Analyses of Vitamin Intake	Higher vitamin A and C intake is associated with reduced risk of glioma instances	[62,63,64,65,66]
Antioxidants	Meta-Analysis of Glioma Patients	Antioxidant effects vary by histological grade of glioma	[67]
Flavonoids	Prospective Study	Reduced risk of glioma instances at higher flavonoid intake	[70]
Vitamin D	Case–Control Study	Serum vitamin D has no impact on glioma instances	[72]
Vitamin D	Meta-Analysis of Mouse Glioma Model and Cell Culture Studies	Reduced tumor growth	[73]
Calcium	Meta-Analysis	Reduced instances of glioma with higher dietary calcium	[106]
Fiber	Meta-Analysis	Reduced instances of glioma with higher dietary fiber	[107]
**Diet Type**	**Study Type**	**Outcomes**	**Reference**
Caloric Restriction	Glioma Mouse Model	Decrease glioma number and size	[79]
Caloric Restriction	Glioma Mouse Model	Decreased tumor growth	[80]
Ketogenic Diet	Glioma Patient Review of Case Studies and Reports	Effect not established	[88]
Ketogenic Diet	Glioma Patient Review of Prospective and Retrospective Studies	Improved survival time and life quality impact not established	[89]
High-Fat Diet (Non-Ketogenic)	Glioma Mouse Model	Activated tumor promoting pathways	[108]
Paleolithic Diet	Case–Control Study	Reduced risk of glioma instances	[99]
Mediterranean, MIND, DASH	Review of Case–Control Studies	Reduced risk of glioma instances	[74]
Mediterranean, DASH	Prospective Study	Increased risk of glioma instances	[103]

## Data Availability

No new data were created or analyzed in this study.

## Abbreviations

The following abbreviations are used in this manuscript:

5-HIAA	5-hydroxyindoleacetic acid
AGR	Albumin/globulin ratio
ALB	Albumin
Bgn	Biglycan
BHB	β-hydroxybutyrate
CAF	Cancer-associated fibroblasts
CAR	Chimeric antigen receptor
CNS	Central nervous system
CR	Caloric restriction
CRP	C-reactive protein
CSF-1	Colony stimulating factor-1
CT	Computed tomography
CTLA-4	Cytotoxic T-lymphocyte-associated protein 4
DASH	Dietary Approaches to Stop Hypertension
DC	Dendritic cell
Dcn	Decorin
DM2	Type 2 diabetes
DNA	Deoxyribonucleic acid
EAA	Essential amino acids
EGF	Epidermal growth factor
ERAS	Enhanced Recovery After Surgery
ESPEN	European Society for Clinical Nutrition and Metabolism
ESR	Erythrocyte sedimentation rate
FFA	Free fatty acids
FODMAP	Fermentable Oligo-, Di-, Monosaccharides, and Polyols
GBM	Glioblastoma
GKI	Glucose ketone index
Hb	Hemoglobin
HFD	High-fat diet
HGD	High-glucose drink
ICB	Immune checkpoint blockade
IDH	Isocitrate dehydrogenase
IFNγ	Interferon gamma
IGF-1	Insulin-like growth factor
IL-10	Interluekin-10
IL-12	Interleukin-12
IL-2	Interlueken-2
KD	Ketogenic diet
LDL	Low-density lipoproteins
MD	Mediterranean diet
MDSC	Myeloid-derived suppressor cells
MGMT	O6-methylguanine-DNA methyltransferase
MIND	Mediterranean-DASH Intervention for Neurodegenerative Delay
MRI	Magnetic resonance imaging
NIH	National Institutes of Health
NK	Natural killer
NRI	Nutritional risk index
OS	Overall survival
OxLDL	Oxidized low-density lipoproteins
PA	Prealbumin
PARP	Poly (ADP-ribose) polymerase
PCV	Procarbazine, lomustine, and vincristine
PD	Paleolithic diet
PD-1	Program death-1
PDGF	Platelet-derived growth factor
PD-L1	Program death ligand 1
PET	Positron Emission Tomography
PGE2	Prostaglandin E2
PNI	Prognostic nutritional index
PUFA	Polyunsaturated fatty acid
RM	Resistant maltodextrin
RNA	Ribonucleic acid
ROS	Reactive oxygen species
SCFA	Short-chain fatty acid
Se	Selenium
TAM	Tumor-associated macrophages
TEE	Total daily energy expenditure
TGF-β	Transforming growth factor-β
TLR	Toll-like receptor
TME	Tumor microenvironment
TMZ	Temozolomide
TTF	Tumor treating fields
VEGF	Vascular endothelial growth factor
WD	Western diet
Zn	Zinc
